# Consumption patterns before and during the COVID-19 pandemic among patients admitted to inpatient drug detoxification treatment: Results of two cross-sectional surveys from 2018 and 2021

**DOI:** 10.3389/fpsyt.2024.1467144

**Published:** 2024-12-16

**Authors:** Patrik Roser, Michael Specka, Udo Bonnet, Thomas Kuhlmann, Stefan Kühnhold, Renate Steinert, Benita Zeiske, Daniel Deimel, Norbert Scherbaum

**Affiliations:** ^1^ LVR-University Hospital Essen, Department of Psychiatry and Psychotherapy, Medical Faculty, University of Duisburg-Essen, Essen, Germany; ^2^ University Hospital of Psychiatry Zurich, Center for Addictive Disorders, Medical Faculty, University of Zurich, Zurich, Switzerland; ^3^ Department of Psychiatry, Psychotherapy and Psychosomatic Medicine, Evangelisches Krankenhaus Castrop-Rauxel, Castrop-Rauxel, Germany; ^4^ Department of Addictive Disorders, Psychosomatic Hospital Bergisch Gladbach, Bergisch Gladbach, Germany; ^5^ Department of Addiction Medicine, LWL Hospital Lippstadt/Warstein, Warstein, Germany; ^6^ Department of Addiction Medicine, LWL Hospital Münster, Münster, Germany; ^7^ Department of Addictive Disorders, Alexius/Josef Hospital, Neuss, Germany; ^8^ Nuremberg Institute of Technology, TH Nuremberg, Faculty of Social Science, Nuremberg, Germany

**Keywords:** COVID-19, opioid use disorder, consumption pattern, gabapentinoids, novel psychotropic substances, route of acquisition

## Abstract

**Background:**

The lockdown measures during the SARS-CoV-2 pandemic could have influenced drug consumption patterns of persons with drug use disorder, especially due to a reduced availability of drugs, an increased consumption of sedating substances as a coping strategy, or a shift to novel psychotropic substances (NPS) associated with an increased drug buying in the internet. In this study, the consumption patterns of people mainly with opioid use disorder entering inpatient drug detoxification treatment were investigated in the same hospitals with the same methods before and during the pandemic.

**Methods:**

At admission, patients were interviewed regarding their consumption patterns using the EuropASI questionnaire. In addition, changes in the routes of drug acquisition were assessed.

**Results:**

In five hospitals in Western Germany, 213 (2021) and 175 persons (2018) were recruited. Sociodemographic data were similar in both cohorts (mean age around 40 years, mainly male, about 50% with migrant background, high unemployment rate). Rates of use of various drugs during the last 30 days were also similar. Differences were detected for gabapentinoids and opioid analgesics (increase >5%) as well as for cannabis (decrease >5%). Current use of NPS was low in both surveys. Only a minority of patients had experiences with drug acquisition in the internet.

**Discussion:**

The pandemic had only a minor influence on consumption patterns and routes of drug acquisition in this sample. It remains to be seen whether the increased use of gabapentinoids and opioid analgesics will continue despite the end of the pandemic.

## Introduction

On March 11, 2020, the World Health Organization (WHO) declared the novel coronavirus (SARS-CoV-2) outbreak a global pandemic (1). As a consequence, the government of the Federal Republic of Germany, in line with many other states, imposed a first strict lockdown from March 22 to May 4, 2020, and a second strict lockdown from December 16, 2020, up to May, 2021, in order to limit the spread of the virus and to relieve the pressure on the health care system (2). The lockdowns particularly included a drastic restriction of social contacts and reduction of international traveling by car, train and aircraft. The restriction of social contacts continued even beyond the two lockdown periods, making it still difficult to gather with other people in the private as well as in the public space.

In the field of addiction medicine, the question on how the pandemic would affect persons with substance use disorder regarding their risk of COVID-19, their mental and physical health, patterns of drug use, and sufficient support by the health care system was discussed (3). It was assumed that pandemic-related stress could lead to a significant increase in substance use, particularly in the use of alcohol and cannabis, in terms of a coping strategy. Secondly, a scenario was discussed that lockdown measures would reduce the availability of illegal drugs sold at public places such as parks or in the surroundings of railway stations. A shortage of heroin and other drugs could in turn lead to more dangerous forms of drug application (intravenous instead of inhalative) and a shift to the increased consumption of still available drugs, especially alcohol. A reduced availability of illegal drugs at the traditional public places could also induce an increased purchase of drugs via the internet, possibly associated with a shift to novel psychotropic substances (NPS) including new synthetic opioids (NSO). Thirdly, a breakdown of the supporting system for people with drug use disorder as a consequence of the lockdown measures as well as of a mass infection among professionals was feared.

Soon after the first lockdown, several cross-sectional surveys, mainly from Europe and North America, showed, at the general population level, mixed results with decreases as well as increases or no changes in alcohol use, but with increased alcohol use being particularly associated with pre-pandemic high-level drinking (4, 5). The results concerning cannabis use among the general population were similarly heterogeneous. While cannabis use patterns among adolescents have not changed markedly, adults showed mixed results with cannabis use having increased, decreased or remained unchanged (6). Of note, depressive symptoms and anxiety during COVID-19 lockdown were significantly associated with increased use of alcohol and cannabis, which might be interpreted as a dysfunctional strategy to cope with negative emotions (7).

Compared to the general population, only limited studies assessed the impact of the COVID-19 pandemic on substance use in patients with pre-existing substance use disorder. In daily cannabis users, the lockdown period in the Netherlands was associated with increased cannabis use, but not with cannabis use disorder severity (8). Similarly, patients with opioid or polysubstance use disorder in ongoing methadone maintenance treatment reported increased substance use during the pandemic in the United States, although the substances were not specified in detail (9). Given the reduced stress tolerance and impaired abilities to cope with stress among people with opioid use disorder (10, 11), it can be assumed that these patients primarily increased the use of sedative substances such as alcohol and benzodiazepines which has been found to be significantly associated with psychological distress in this population (12, 13).

Mixed results were obtained from two Spanish cross-sectional studies in patients under outpatient treatment for substance dependence, particularly for alcohol, cannabis and opioid use disorder (14, 15). With specific regard to opioid use during the COVID-19-related lockdown, one of the two studies reported on an only small proportion of patients who decreased their opioid use compared to the time before the lockdown, whereas the opioid consumption pattern of the vast majority of the respective population remained unchanged (14). On the other hand, the second study showed an increase of opioid use in almost half of the patients with opioid use disorder during the lockdown, compared to before, and markedly less patients who decreased their opioid use in the same time period (15). However, despite the few aforementioned studies with relatively small sample sizes and, at least in part, contradictory findings, there is still limited evidence of the impact of the COVID-19 pandemic on the drug consumption of opioid dependent persons (16).

In this study, the drug consumption pattern of people with substance use disorder was investigated during the COVID-19 pandemic. In contrast to other studies investigating the impact of the pandemic on drug use (8, 9, 14, 15), baseline data from the pre-pandemic were available. In 2018, the research team carried out a survey investigating consumption patterns of people with drug use disorder entering inpatient detoxification treatment in psychiatric hospitals in Western Germany (17). In 2021, the same survey was again carried out in five out of the eight hospitals participating in the previous survey. The following questions were addressed in the investigation during the pandemic:

Did the drug consumption pattern change.

a) as a potential consequence of a reduced availability of illegal drugs, e.g., increase of intravenous application, shift towards legal drugs such as alcohol and benzodiazepines, or shift towards NPS including NSO?

b) as potential coping strategy with the pandemic-related stressors, e.g., increase in the use of sedating substances such as alcohol or benzodiazepines?

In addition, routes of drug acquisition were investigated assuming that a reduction of drug trafficking at usual public spaces due to the lockdowns would increase the acquisition of drugs in the internet.

## Methods

### Study design

Five psychiatric hospitals in the federal state of North Rhine Westphalia in the Western part of Germany participated in this prospective, cross-sectional multicentre study. Data were collected at two time periods: (1) during the year 2018 (before the COVID-19 pandemic) and (2) between March and September 2021 (during the COVID-19 pandemic). During both time periods, the participants were recruited for the study at admission to inpatient drug detoxification treatment.

Participants had an interview which included questions about their past and present drug use as well as sociodemographic characteristics (i.e., age, gender, migration background, relationship status, living with children and employment status). The interview was carried out by medical staff members of the respective institutions and was based on the European Addiction Severity Index (EuropASI, German version (18)). In the section on consumption history (lifetime prevalence, last 30-days prevalence) of various drugs such as alcohol, amphetamines, benzodiazepines, cannabis, cocaine, and heroin, items regarding different new psychoactive substances (NPS; e.g., synthetic cannabinoids, synthetic stimulants, herbal drugs), novel synthetic opioids (NSO; e.g., carfentanyl, U-47700), gabapentinoids and opioid analgesics were already added in 2018. Those patients who were included during the second study period (during the COVID-19 pandemic) were additionally asked to report on how the pandemic has affected their use of each substance (i.e., unchanged, variable, reduced, increased). Finally, they should indicate whether they made use of specific routes of drug acquisition before or, for the first time, during the pandemic (i.e., drug acquisition via internet, darknet, messenger services and/or home delivery).

### Study sample

All patients who were admitted to inpatient drug detoxification treatment during the two study time periods and met the eligibility criteria were invited to participate in the study. Prior to inclusion, they were informed in detail about the study aims and procedures, particularly the pseudonymization and protected storage of their data. All participants gave their written informed consent.

Patients were eligible to participate in the study if they were at least 18 years of age and diagnosed with dependence from amphetamines, cannabis, cocaine and/or opiates, according to ICD-10 diagnostic criteria. Exclusion criteria were as follows: insufficient understanding of the German language; significant cognitive impairments due to intoxication or withdrawal syndrome (patients could be included later during their treatment when intoxication or withdrawal symptoms had ceased); and acute episode of a severe comorbid mental disorder (e.g., psychosis). Patients could withdraw from study participation at any time and without any negative consequences.

### Data analysis

Statistical analyses were carried out by using descriptive statistics indicating absolute frequencies and percentages. Group comparisons of sociodemographic and substance use characteristics were calculated by using asymptotic Chi^2^ test for categorical and Welch’s t-test for continuous variables. Data were analyzed with IBM SPSS Statistics for Windows, Version 25.0 (Armonk, NY: IBM Corp.).

## Results

### Study participants

In 2021, five out of eight hospitals already participating in the survey in 2018 took part again in the survey. In these hospitals, 175 patients had taken part in the survey in 2018, whereas 109 patients had to be excluded due to various reasons (n=39: insufficient understanding of the German language, n=43: significant cognitive, psychiatric or substance-related problems; n=18: refusal of participation; n=9 refusal to complete the interview). In 2021, 213 patients could be interviewed at the same detoxification wards, whereas 120 patients had to be excluded due to various reasons (n=57: insufficient understanding of the German language, n=31: significant cognitive, psychiatric or substance-related problems; n=26: refusal of participation; n=6 refusal to complete the interview). The recruitment rates were similar in both years (2021: 61.6% vs. 2018: 64%).

As can be seen in **Table 1**, sociodemographic characteristics were similar in both cohorts: the mean age was around 40 years, mainly male, more than half of them had a migration background, lived in a steady relationship and were currently in opiate maintenance treatment (51% and 61%, respectively), the unemployment rate was over 80%. In both samples, the most often (in more than 80%) diagnosed substance-related disorder was opioid dependence (see **Table 1**). Also, the prevalence of cannabis dependence, cocaine dependence and benzodiazepine dependence were similar in both samples. Alcohol dependence was more often diagnosed in 2021 (43%) than in 2018 (35%). Amphetamine dependence was only little prevalent in both, however even less in 2021 (3.9%) than in 2018 (14%).

**Table 1:** Sociodemographic and substance use characteristics of the two samples.

	2018 (n=175)		2021 (n=213)		Group comparison^1^
	%	n	%	n	p
Age					
Mean (SD)	38.1 (8.9)		40.2 (9.9)		0.27
Gender					
Male	83.2%	145	75.5%	161	0.10
Female	16.8%	30	24.5%	52	
Migrant background					
Self or one parent foreign born	54.9%	96	55.0%	117	0.98
No migrant background	45.1%	79	45.0%	96	
Stable relationship					
Yes	57.7%	101	51.9%	111	0.25
No	42.3%	74	48.1%	102	
Children in household					
Yes	11.4%	20	12.2%	26	0.64
No	88.6%	155	87.8%	187	
Currently employed					
Yes	15.4%	27	11.3%	24	0.31
No	84.6%	148	88.7%	189	
In opioid maintenance treatment					
Yes	51.0%	89	61.0%	130	0.044
No	49.0%	86	39.0%	83	
Substance-related diagnoses (multiple diagnoses possible)					
Opiates	83.0%	145	84.1%	179	0.81
Cannabis	35.1%	61	33.8%	72	0.75
Cocaine	36.6%	64	37.2%	79	0.95
Alcohol	35.4%	62	43.0%	92	0.12
Benzodiazepines	24.0%	42	27.1%	58	0.55
Amphetamines	13.7%	24	3.9%	8	< 0.001
Lifetime use (any use)					
Alcohol	86.9%	152	91.1%	194	0.22
Amphetamines	69.7%	122	72.8%	155	0.67
Benzodiazepines	64.6%	113	69.0%	147	0.42
Cannabis	89.1%	156	90.6%	193	0.57
Cocaine	90.3%	158	89.7%	191	0.84
Gabapentinoids	44.0%	77	57.7%	123	0.023
Heroin	87.4%	153	91.5%	195	0.11
Hallucinogens	53.7%	94	54.0%	115	0.96
Inhalants	16.6%	29	21.1%	45	0.26
MDMA/Ecstasy	62.3%	109	64.8%	138	0.78
Methamphetamine	17.1%	30	18.8%	40	0.71

^1^Asymptotic Chi. ^2^test, except Welch’s t-test for “age”.

### Current and life-time use of different drugs

**Figure 1:**
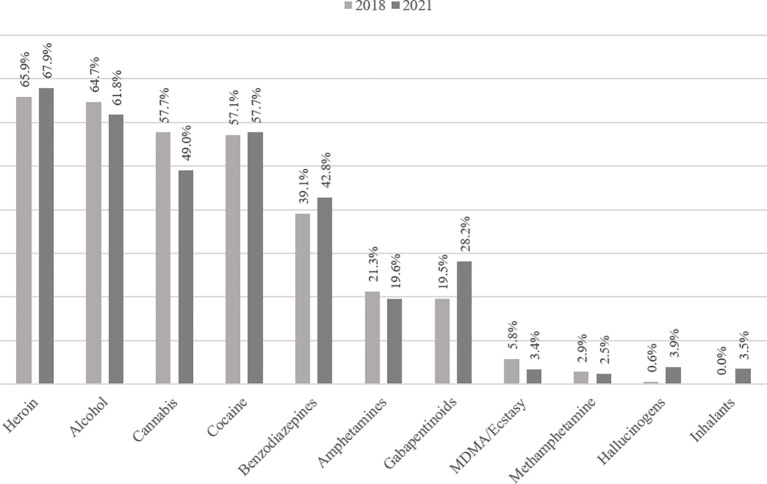
Current use of drugs (last 30 days prior to admission) before and during the COVID-19 pandemic.

A high percentage of patients had high lifetime experience with standard drugs (heroin, alcohol, cannabis, cocaine, benzodiazepines, amphetamines, MDMA/ecstasy, hallucinogens, sniffing substances, and gabapentinoids). Regarding the question of a change in the consumption pattern, the current use (last 30 days before admission) of drugs is of more importance (see **Figure 1**). For most drugs, the current level of use was similar between the two samples. However, there was an increase in the percentage of patients reporting a current use of gabapentinoids (from 19.5% to 28.2%) and a decrease of patients reporting a current use of cannabis (from 57.7% to 49.0%). Current intravenous use of one or more substances was reported by 35.1% (2018) and 37.9% (2021), respectively.

**Figure 2:**
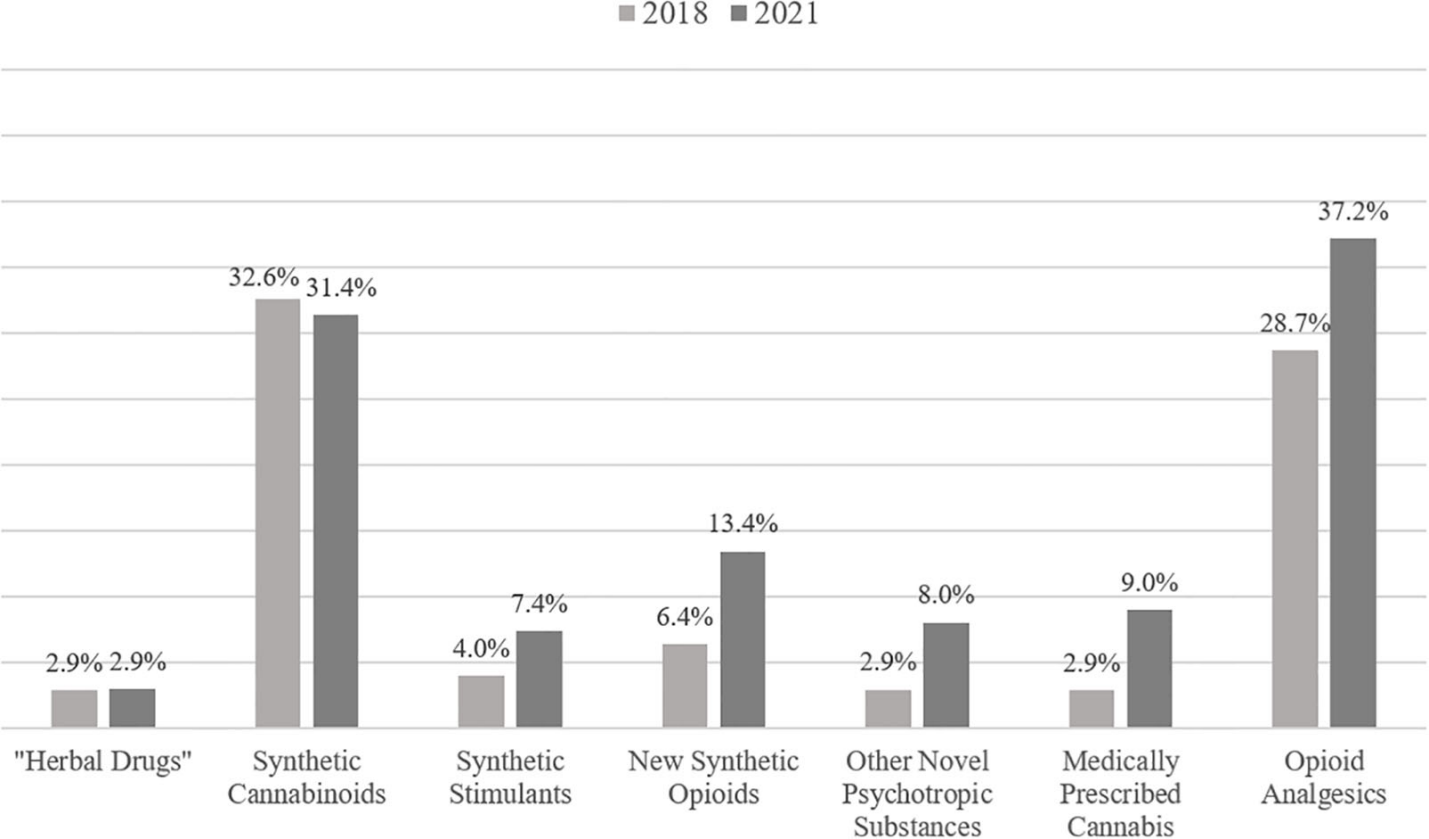
Lifetime use of different novel psychotropic substances, new synthetic opioids, medically prescribed cannabis and opioid analgesics before and during the COVID-19 pandemic.

**Figure 3:**
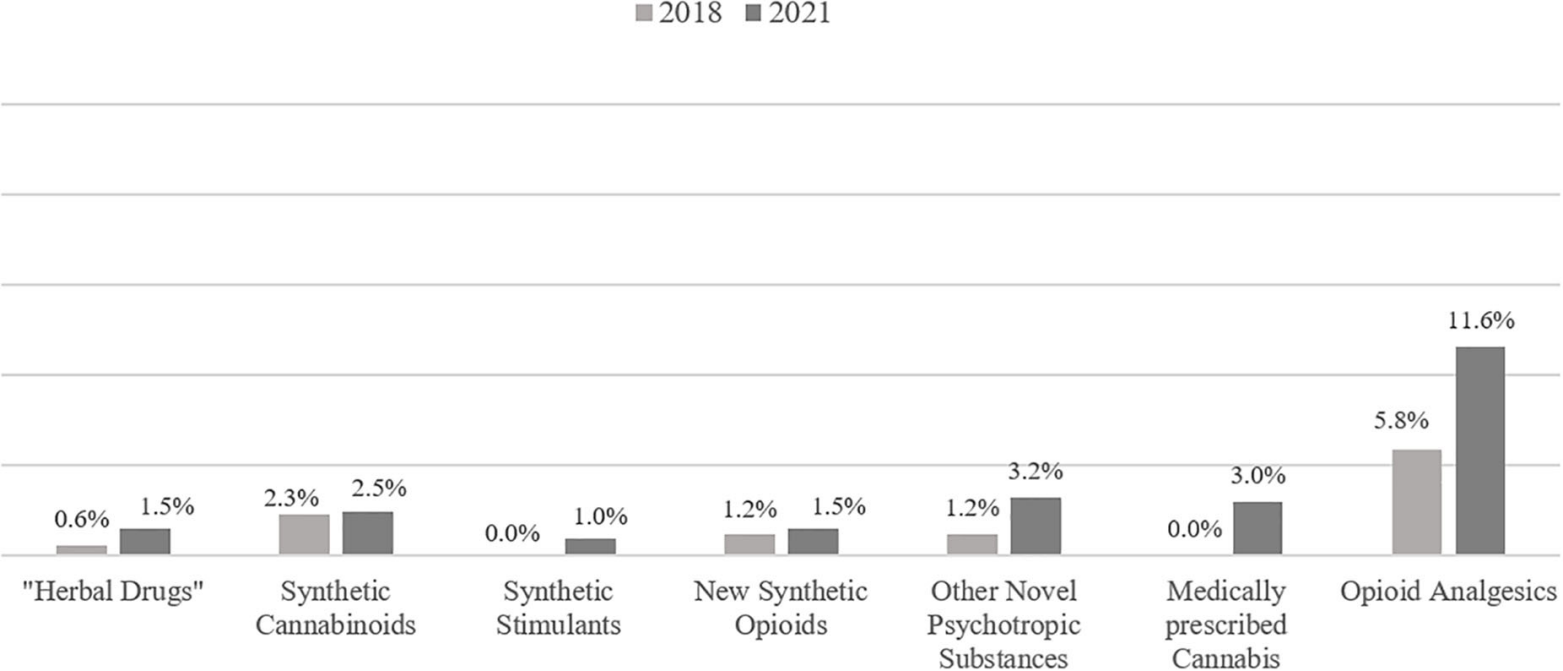
Current use of different novel psychotropic substances, new synthetic opioids, medically prescribed cannabis and opioid analgesics before and during the COVID-19 pandemic.

In addition, the use of drugs which could be used as alternative of traditional drugs, such as different NPS (e.g. synthetic cannabinoids, synthetic stimulants), NSO, medically prescribed cannabis and opioid analgesics was analyzed. The lifetime prevalence of the use of these drugs (see **Figure 2**) was especially high (about a third of patients) for synthetic cannabinoids and opioid analgesics. An increase of the percentage of patients reporting the lifetime use of the respective drugs of at least 5% was documented for NSOs, other NPS (e.g., 2-CB, methaqualone), medically prescribed cannabis, and opioid analgesics. Regarding current use, there was an increase in the use of opioid analgesics from 5.8% up to 11.6%. Regarding all other substances, current use was only reported from small minorities of both samples (0% up to 3.3%, see **Figure 3**).

### Subjective changes in substance use due to the COVID-19 pandemic

The vast majority of the participants of the second study period stated that the COVID-19 pandemic did not markedly affect their substance use patterns (results not shown in detail). Analyzing the data of self-reported lifetime users, the highest proportions of increased use were seen for alcohol (16.4% of lifetime alcohol users), benzodiazepines (13.0%), gabapentinoids (11.6%), and heroin (11.0%) (see **Table 2**).

**Table 2:** Proportion of lifetime users of the respective substances or substance classes who reported decreased/increased use due to the COVID-19 pandemic. Substances were ordered according to the proportion of increased use, in descending order.

	Unchanged/ fluctuating/ not specified	Decreased	Increased	n
Common drugs of abuse				
Alcohol	82.5%	1.1%	16.4%	183
Benzodiazepines	84.8%	2.2%	13.0%	138
Gabapentinoids	86.6%	1.8%	11.6%	112
Heroin	80.1%	8.9%	11.0%	191
Cannabis	84.6%	7.1%	8.2%	182
Cocaine	86.1%	6.1%	7.8%	180
MDMA/Ecstasy	95.8%	3.4%	0.8%	118
Amphetamines	90.2%	5.3%	4.5%	133
Hallucinogens	97.0%	2.0%	1.0%	101
Methamphetamine	96.9%	3.1%	0.0%	32
Inhalants	96.9%	3.1%	0.0%	32
Alternative substances				
Medical cannabis	90.9%	0.0%	9.1%	22
Synthetic opioids	87.1%	6.5%	6.5%	31
Opiate analgesics	90.8%	3.9%	5.3%	76
Synthetic cannabinoids	98.5%	1.5%	0.0%	65
Synthetic stimulants	95.0%	5.0%	0.0%	20
Other NPS*	100.0%	0.0%	0.0%	37
“Herbal drugs”	91.7%	8.3%	0.0%	12

*NPS, Novel Psychoactive Substances.

### Routes of drug acquisition before and during the COVID-19 pandemic

**Figure 4:**
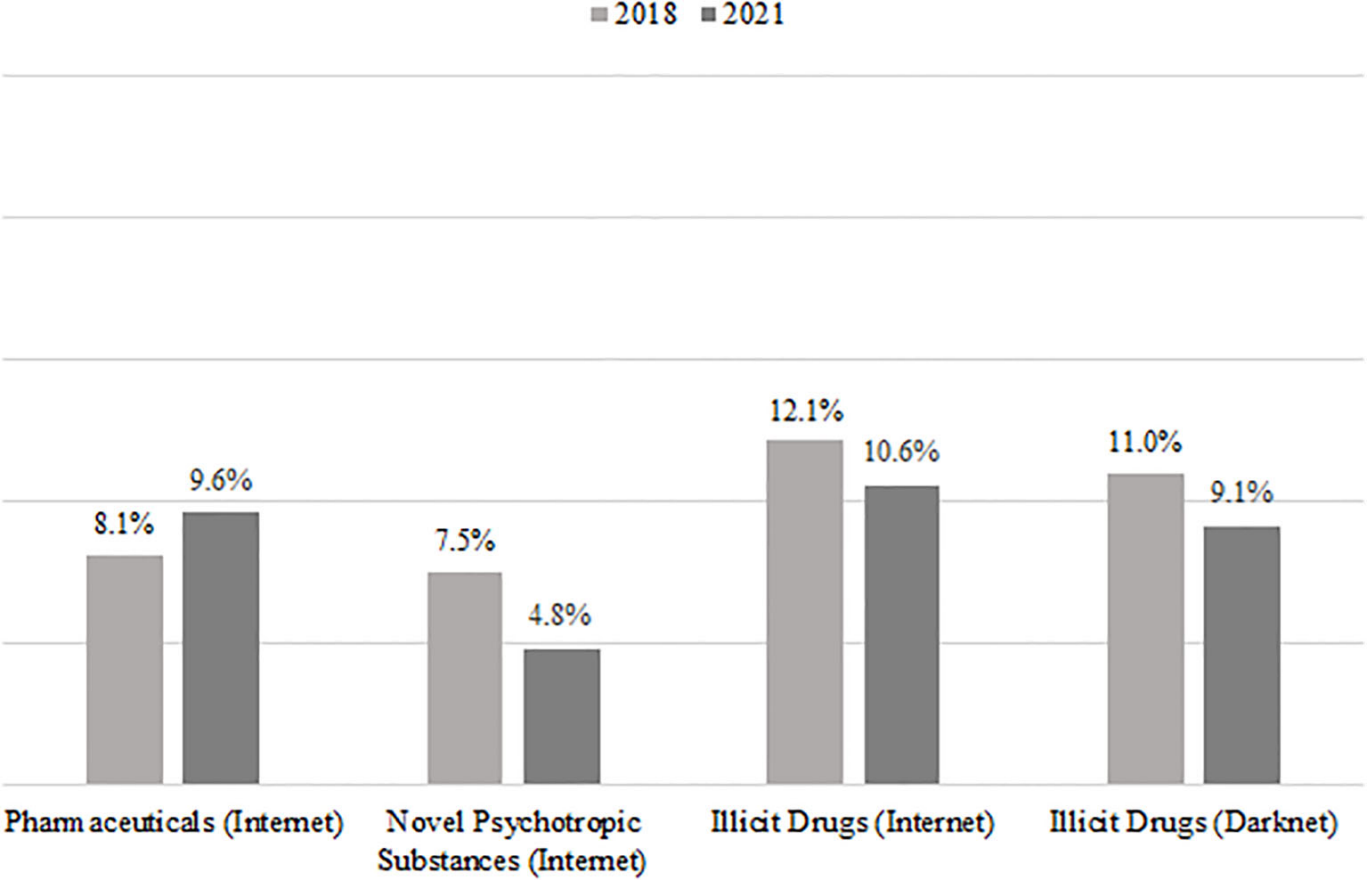
Routes of drug acquisition before and during the COVID-19 pandemic.

Only a small minority of patients reported in 2018 as well as in 2021 that they ever ordered pharmaceuticals (2018: 8.1%; 2021: 9.6%), NPS (7.5% vs. 4.8%) or illicit drugs (12.1% vs. 10.6%) in the common internet, or illicit drugs in the darknet (11.0% vs. 9.1%) (see **Figure 4**).

In the second survey, patients were also asked whether they used special services during the pandemic for the first time. During lifetime, 25.0% had ordered illicit drugs via messenger services, including 4.8% who had done this for the first time during the pandemic (another 4.8% did not clearly indicate the time period); 14.4% had received drugs through home delivery services, including 1.9% who had started this route of acquisition during the pandemic (another 4.3% did not indicate the time period). All other routes of acquisition were only used by small minorities and their use did only increase by single persons during the pandemic.

## Discussion

This study was carried out to investigate the impact of the COVID-19 pandemic on the consumption patterns of people with drug dependence. For this aim, drug (mostly opioid) dependent persons in Western Germany were investigated at admission to inpatient detoxification treatment in five psychiatric hospitals using a standardized questionnaire. Apart from a few additional questions related to the pandemic, the same survey was carried out in the same hospitals before (2018) and during the pandemic (2021). According to our data, the drug consumption patterns differed only to a small extent, especially regarding alcohol that was more often diagnosed as comorbid substance-related disorder, as well as gabapentinoids and opioid analgesics that were more often reported as currently used. Despite an increase in the lifetime use of NPS and NSO, there was no relevant shift to the use of NPS and NSO during the pandemic, as indicated by mostly unchanged prevalence rates of current use and no substantial differences in the subjective evaluation of changes in consumption patterns. The percentage of persons reporting current intravenous application of drugs was very similar. Only a minority used the internet for drug acquisition, whereas the use of messenger services was, with about 25% of the patients, already widespread before and increased during the pandemic.

The most important argument for the hypothesis that the pandemic would lead to changes of drug consumption patterns was the assumption that, especially due to the extensive and long-lasting lockdown measures, the availability of illegal drugs would be reduced. However, according to own data collected in opioid maintenance clinics and low-threshold institutions such as a heroin-prescription clinic and a café for people with drug dependence, the availability of illegal drugs was not markedly decreased in 2020 (19). This estimation is in accordance with observations that the amount of drug seizures by the police as well as other indicators did not support the expectation of a generally reduced availability of illegal drugs in the European Union (20, 21). In this context, it has to be noted that the lockdowns reduced private (international) travelling, but not the transport of goods, e.g., by trucks or ships.

As far as illegal drugs are sufficiently available and payable, there is no need for people with drug dependence to change consumption patterns, e.g., to shift to an increased consumption of NPS and NSO. Especially regarding synthetic cannabinoids, both before and during the pandemic, a high lifetime prevalence of use was reported by about one third of the patients, in contrast to a rather low current prevalence of use of less than 3%; it appears that the majority of the participants had an experimental use of synthetic cannabinoids in the past, but did not continue to use them, presumably due to aversive effects of these substances (17). In addition, if drugs are available in traditional ways, there is also no pressure to use alternative ways of drug acquisition via the internet. It has to be noted here that the investigated study sample consists of a socially disadvantaged group with a high unemployment rate and presumably (if any) only low financial resources. Therefore, it can be assumed that, in this specific group, purchases of any goods via the internet is of only minor importance. This might be different in other groups of drug users; for example, the probability of online purchase of illicit drugs in the general population was associated with higher education and higher income (22), while clinical samples such as those presented in this study generally show lower education and lower income. However, even in the socially disadvantaged group of this study, the ordering of drugs by messenger services as well as home delivery were reported not only by single persons but by a quarter of the patients. This might indicate that even in this group new ways of interaction do change the acquisition of drugs.

A second expectation relates to a putative increase of the use of sedating substances in order to cope with pandemic-related stressors. Here, we found some indicators, such as the increase of the diagnosis of comorbid alcohol dependence, the increase of the current use of gabapentinoids, and the subjective evaluation by a subgroup of patients in 2021 that they had increased their use of alcohol, benzodiazepines, and gabapentinoids. In this context, it has to be considered that one of the major problems of people with opioid dependence is their reduced capacity to cope with stress, e.g., on the basis of traumatic experiences during childhood as well as deficits in socialization and education (23). The pandemic was associated with stressful experiences, especially for people who use illicit drugs. They were declared as a risk group for infection with SARS-CoV-2 and a severe course of COVID-19, they were at risk to reduce even more their anyway rare social contacts, and the maintenance treatment was at risk if there would be an outbreak among the professionals of their treatment office. Actually, maintenance treatment was reliably available in North Rhine during the pandemic (24). Moreover, the introduction of COVID-19 vaccines and the national pro-vaccination campaigns may have created additional feelings of anxiety and fear based on doubts about the vaccines’ efficiency and safety (25). In this respect, it can be assumed that vaccination-related psychological stress may also have contributed to the increased use of sedating substances as a coping and self-medication strategy, especially alcohol (26).

However, the number of patients who reported a current use of cannabis within the last 30 days decreased, although the prevalence rate of cannabis use disorders and the subjective evaluation of changes in cannabis use patterns did not markedly differ from pre-pandemic findings. It remains speculative whether some patients might have switched from cannabis to other sedating substances, e.g., alcohol or benzodiazepines, due to reduced availability (19), or to medically prescribed cannabis which lifetime and current use increased markedly during the pandemic. Nevertheless, a recent review reported no significant impact of the COVID-19 pandemic on cannabis use patterns (6).

In contrast to the developments in the use of sedating substances, the problematic use of psychostimulants decreased significantly during the pandemic, as indicated by a lower prevalence of amphetamine use disorders and reduced current use of MDMA/ecstasy and methamphetamine in 2021. Similar findings were obtained by recent studies from Europe (27) and Australia (28). Most probably, the restriction of social interactions contributed to the decrease in the consumption of psychostimulants which are most commonly used in social contexts and recreational settings including the party and club scene.

### Limitations and strengths

The results of this study are based on the self-report of patients and there were no systematic evaluations using urine drug screens. However, in the context of a detoxification treatment, patients´ reports are considered reliable as they are interested that their withdrawal symptoms are sufficiently alleviated by medication. For the medication plan, the physicians need a valid history on current consumption patterns. In addition, a strength of this study is the fact that the survey was carried out in a prospective design at two times with a baseline observation already before the onset of the pandemic. Therefore, the analysis of the consumption pattern in 2021 is not based on the retrospective report of patients about drug consumption during the years before the pandemic. Quite the opposite, the most important data relate to patients´ report about drug consumption during the limited time of the last 30 days.

In an observation study with two points in time (here: 2018 and 2021), it is difficult to evaluate whether changes between these points are related to specific events during the observation period (here the pandemic). There were first anecdotal reports on the misuse potential of pregabalin more than 10 years ago (29) and in the following years abuse of gabapentinoids was observed as an increasing problem, especially in opioid dependent persons (30). However, already in 2018 almost 20% of the patients of this sample reported the use of gabapentinoids in the last 30 days before admission. Therefore, it is difficult to evaluate the further increase of the use of these substances as a result of the pandemic. In a similar way, the increase of the use of opiate analgesics is difficult to interpret. Therefore, it might have occurred independently from the pandemic just as a change in the preference of opioids by this group. The opioid epidemic in the USA with a high importance of the use of opioid analgesics started long before the onset of the pandemic (31).

## Data Availability

The raw data supporting the conclusions of this article will be made available by the authors, without undue reservation.
